# Editorial Comment: Abiraterone in “High-” and “Low-risk” Metastatic Hormone-sensitive Prostate Cancer

**DOI:** 10.1590/S1677-5538.IBJU.2021.01.08

**Published:** 2020-11-18

**Authors:** Felipe Lott

**Affiliations:** 1 Instituto Nacional do Câncer Rio de JaneiroRJ Brasil Instituto Nacional do Câncer - INCA, Rio de Janeiro, RJ, Brasil

Hoyle AP ^1^, Ali A ^2^, James ND ^3^, Cook A ^4^, Parker CC ^5^, de Bono JS ^5^, et al.

## COMMENT

Two randomised trials has established the use of Abiraterone (AA) as an alternative standard of care to Docetaxel treatment in men with metastatic Hormone Naïve Prostate Cancer (mHNPC) with “high risk” or high volume disease (1, 2). Uncertainty exists in the benefit of AA in “low risk” M1 disease.

This trail uses the STAMPEDE platform (3) design, randomizing 1:1 for use of AA + androgen deprivation therapy (ADT) vs ADT alone.

There were a 34% survival benefit in the AA group also in the “low risk” mHNPC although the number needed to treat to prevent one death was 4 times higher in “low risk” group compared with the “high risk”.

This trial results support the use of abiraterone in mHNPC irrespective of “risk” or “volume.
